# Notes and News

**Published:** 1887-04-09

**Authors:** 


### Notes and News.

COLLECTED AND COMMUNICATED.

HER Majesty the Queen has sent a cheque for £100 to the Queen's Hospital at Birmingham.

MR. W. B. KIRKWOOD has just presented a valuable piano to the British Home for Incurables, Putney.

THE special fund to raise £100,000 for Guy's Hospital is to be assisted by a dramatic performance at the Novelty Theatre, on Thursday, 21st April. The performance is to be under distinguished patronage. The Fund now amounts to rather over £56,000, not including conditional donations. There are about 130 beds at present closed at the hospital.

THE annual festival dinner of University College Hospital is to be held on Wednesday, 27th inst., at the Criterion, Piccadilly. The Right Hon. G. J. Goschen, M.P., has promised to preside on the occasion.

AN American journal states that a doctor, carrying on practice in one of the principal streets of Pittsfield (New York), informs patients, on his door-plate, that he is to be found " hear on fridys and Saterdys only."

MR. ROBERT FREWER, the Secretary of the Hospital Saturday Fund, stated at the last meeting of the delegates that, as the result of the first year's work of the Surgical Appliance Committee, 540 patients had been supplied with surgical instruments at an average cost of ten shillings, the attendances recorded being 695.

THE Board of the Children's Hospital in Western Bank, Sheffield, have decided, in compliance with a resolution passed by the governing body in February, to erect additional buildings as soon as the requisite funds for this very desirable object have been raised. Indeed, instructions for the preparation of plans have already been issued, for the Board believe that no difficulty will be experienced in obtaining the necessary sum of £1,000, several benevolent and generous friends having promised assistance. It is intended to name a cot in the hospital after every donor of £100.

MR. JAMES S. BLYTH, the Secretary of the Royal Free Hospital, where the brave Finchley policeman is now lying, has opened a fund to enable the committee to set up the constable in a suitable business. Contributions should be sent at once to Mr. Blyth, at the Hospital, Gray's Inn Road, W.C.

MR. G. A. SALA, who takes a warm interest in the Royal Hospital for Children and Women, Waterloo Bridge Road, has kindly promised to give, in its aid, a lecture on his recent travels in Australia and New Zealand, at St. James's Hall, on 11th May. The lecture is to be honoured by the patronage of Prince and Princess Henry of Battenberg.

THE appeal of the East London Hospital for Children, to enable the Board of Management to effect some very needful and pressing improvements, has been met by one or two generous offers. One friend has agreed to give £200 if four others will subscribe £200 each, or eight others £100 each, making £1,000 in all. Another has offered £50, if nine more will each contribute a similar sum, making £500. Who will respond?

A LECTURE will be given on Friday evening, the 15th April, at the Midwives' Institute and Trained Nurses' Club, 15, Buckingham Street, Strand, by Dr. Henry D. Waugh, on "The Nursing of Cases of Difficult Breathing." Lecture to commence at 7.45.

AFTER a good deal of opposition, it has been finally determined to erect a hospital for the southern district of the City of Glasgow, as a memorial of the Queen's Jubilee. It is to be hoped that the new hospital will not be the means of lessening the incomes of the existing charities of Glasgow. In response to a communication from the Lord Provost, the Home Secretary has replied on behalf of the Queen,—" Her Majesty approves of the hospital at Glasgow, as to which application has been made, being styled the Victoria Infirmary."

THE annual meeting of the subscribers of the Shropshire and Wales Eye, Ear and Throat Hospital at Shrewsbury was held early in March, the Mayor (Alderman G. B. Lloyd) being in the chair. The 67th annual report, which was presented by the committee, stated that although there was a satisfactory increase in the annual subscriptions, yet the total receipts for the year had decreased as compared with 1885. This was caused by the falling off in the legacies and donations received during the period under review, and also by a marked diminution in the amounts obtained from Hospital Saturday collections in Shrewsbury and Wellington. The number of patients admitted during the year was 1,512, of whom 208 had been in-patients, there having been an average per week of nineteen occupied beds. Regret was expressed at the death of Dr. Andrew, whose exertions, since his appointment as honorary surgeon in February 1862, had been unwearying in the promotion of the success and prosperity of the hospital. An able successor to him has been found in Dr. William Charnley, formerly surgeon to the Central London Ophthalmic Hospital.

The following vacancies are announced this week, the amount of the salary being placed in each case after the name of the institution :—

**Honorary Medical Officer.**

Chelsea Hospital for Women. Physician, duly-qualified.

**Resident Medical Officers.**

Birmingham Queen's Hospital. Obstetric and Ophthalmic House Surgeon, qualified.
Bradford Infirmary. House Surgeon, doubly-qualified.—£110.
Gloucester County Asylum. Third Assistant, fully-qualified.—£105.
New Hospital for Women, Marylebone Road. Medical woman as Clinical Assistant, doubly-qualified.
Paddington Green Children's Hospital. House Surgeon.—£80.

**Non-resident Medical Officers.**

Dental Hospital of London. Dental Surgeon, licentiate.
Poplar Union. Duly-qualified.—£130.

**Trained Nurses.**

Colchester Hospital. One.—£25.
Frome Nurses' Home. One.—£20 to £25.
Greenwich Seamen's Hospital. One.—£20.
Newcastle-upon-Tyne, St. Mary Magdalene Home. One. Night and day duty alternately.—£20.
Southport Infirmary. One. Night.—£20.
West London Hospital. Assistant Nurse.—£24.

**Probationers.**

Norwich, Jenny Lind Hospital. One. Terms, £1 per week.
West London Hospital. Several.—£12.

---

The Poet Laureate, in his Ode in commemoration of Her Majesty's Jubilee, appearing in this month's number of *Macmillan*, has done well to remind the rich of their duties in regard to charity. "Give your gold to the Hospitals," sings Tennyson. Never was there an occasion when our hospitals stood in more pressing need of help, and never was there a more fitting occasion than this year of Jubilee for those that "wanton in affluence" to give to these institutions. "Give your gold to the Hospitals" is the Laureate's counsel to the rich, and it is good. Some of our hospitals might appropriately adopt the line in their appeals to the public; indeed, we see the London Hospital has already done so.

---

The progress made by the North-West London Hospital during the comparatively short period of eight years is certainly very gratifying. The little hospital was established in 1878, in the Kentish Town Road. After three or four years, a new wing, christened the "Helena," was opened in July 1883 by Princess Christian, the patron of the institution, extending the accommodation to forty-five beds. The annual report for the past year shows that 602 in-patients were admitted, while 33,687 cases were treated in the out-patient department. The receipts amounted to £3,477, including £100 from the United Friendly Societies, and four representatives of these bodies have been made honorary life governors. The North-West London Hospital is fortunate in possessing some very warm and enthusiastic friends, through whose exertions the funds last year were very greatly assisted. The hospital, though now a general one, has special provision for the treatment of sick children. Last year two new cots were presented to the hospital, the "Lily," by Miss M. Pope, and lately the "Violet," by Miss J. Leney. The committee have secured a large house adjoining the institution to meet the increasing wants. The garden of the house will afford a pleasant resort in the summer months for convalescent patients.

The Royal Hospital for Children and Women in the Waterloo Bridge Road has an annually recurring difficulty in making "both ends meet," consequent on its ordinary income falling short of its expenditure by about £2,000. Owing to the necessary work of cleansing and repainting the wards, and other exceptional causes, the hospital fell during the past year £800 into debt. Some of our medical charities are beginning to think that the Jubilee appeals are being a little overdone, but not so with the Royal. At the annual meeting, at which the Lord Mayor presided, it was unanimously resolved to lift the hospital out of its present difficulties by raising a Jubilee Fund of £1,000. For this purpose £355 was raised in the room, including a special donation of ten guineas from the Lord Mayor.

---

The Board of Governors of the Wolverhampton and Staffordshire General Hospital was held on the 8th March, Lieutenant-Colonel Thorneycroft (the chairman of the institution) presiding. The thirty-eighth annual report set forth that the number of in-patients treated during 1886 amounted to 1,956, of whom 1,598 were discharged cured or relieved. The number of out-patients was 11,858, an increase of 80 as compared with the preceding twelve months. The statement of accounts showed a falling off of nearly £300 in the receipts, owing to a decrease in the annual subscriptions and the Hospital Sunday and Saturday collections; whilst a less sum than usual had been received for extra tickets owing to cases of fever, occurring in the town, being treated at the new borough infectious hospital. Seventy-three women and children had been admitted to the St. Catherine Convalescent Home at Penn, and had been greatly benefited thereby.

---

The authorities of the Royal Infirmary, Bristol, are about to try the experiment of putting new floors to their wards on the top of the old ones. The latter have, through long use and frequent washing, become very uneven and full of cracks, which cause them to be regarded as unhealthy, and likely receptacles for the germs of infectious diseases. It is therefore proposed, to save the expense of entire reflooring, to lay oak or teak floors on the existing ones, and have them polished and waxed in future, and so avoid washing. We cannot commend the proposal, and our experience leads us to believe that blood-poisoning and its attendant evils are likely to result in consequence. Indeed, we feel it incumbent upon us to say that the surgical staff of the Royal Infirmary ought to resign, rather than to permit this dangerous proposal to be carried out in the case of the operation and surgical wards. Should any patient admitted to the infirmary with an open wound, or who has been operated upon in the wards after the new floors have been placed upon the old ones, die, or even suffer from blood-poisoning, his friends would no doubt have good grounds for an action for damages. Such so-called economies as that proposed at the Bristol Infirmary ought to be repudiated by the medical staff and the great majority of the governors. This is a case where the local press ought to interfere in the interests of the patients and the public.

THE following sums have been recently received by the various Medical Charities, the name of the donor or the source from which they were obtained being given in each instance after the name of the Institution :—

**Donations.**

Charing Cross Hospital. Hon. A. G. Tollemache, £100.
Dental Hospital of London. Mr. Chas. Morley, 10 guineas. Hon. A. G. Tollemache, £25.
British Home for Incurables, Putney. Mr. C. C. Gooch, 10 guineas. Mr. Graham Hastings, Q.C., 10 guineas.
Royal Ear Hospital, Frith Street, Soho. Duke of Bedford, 10 guineas.
London Homœopathic Hospital and Medical School. Mr. Henry Tate, £100 (towards the new ward).
North London Hospital, Gower Street. Rt. Hon. Lord Justice Fry, £50.
West London Hospital. Messrs. L. Cowan and Sons, 10 guineas. Brentford Union, 20 guineas. Receiver of the Metropolitan Police District, 10 guineas. La Société Anonyme de l'Hippodrome, £20.
Hospital for Consumption, St. Leonards-on-Sea. Mercers' Company, 25 guineas.
Royal Hospital for Children and Women, Waterloo Bridge Road. Mr. J. S. Morgan, £100.
British Home for Incurables. Mr. H. W. Prescott, 10 guineas.
Great Northern Central Hospital. Maintenance Fund: Charles H. de Loysa, £50; Building Fund: H. D. Tufnell, £500; F. Reckitt, £150; E. Homan, £105; W. and A. Reynolds, £105; Watson Surr, £100; Wm. Candery, £100; W. J. Johnstone, £100; W. Piper, £52 10*s.*; Lady Wantage, £50; James Hughes, £52 10*s.*; Misses Fletcher, £50.

**Bequest.**

Great Northern Central Hospital. Mr. G. W. B. Bentinck, £1,000.

**Entertainments.**

Victoria Hospital for Children, Chelsea. Proceeds of Entertainment given by the pupils of the Earl's Court Schools, per the Rev. W. H. Bruce, £11 13*s.* 3*d.* (making a total of £36 contributed by the pupils of this school during the past three years).

**Legacies.**

Liverpool Royal Infirmary. Mrs. Carew, of Mytton Hall, Salop, £1,000.
British Home for Incurables, Putney Trustees of Berman's Charity, 10 guineas.
Henshaw's Asylum for the Blind, Manchester. Mrs. Elizabeth Salisbury Heywood, late of Bowdon, £200.
St. Mary's Hospital and Dispensary, Quay Street, Manchester. Mrs. Elizabeth Salisbury Heywood, late of Bowdon, £200.

**Collections.**

West London Hospital. Offertory at Fulham Parish Church, £14 3*s.* 6*d.*

---

THE Midwives' Institute and Trained Nurses' Club, 15, Buckingham Street, Strand, deserves encouragement and support in every way. We publish in another column the first part of a lecture which Dr. Stretch Dowse delivered to the members a fortnight ago, and which will well repay perusal. This and former lectures have been of great practical value and interest, and for this reason the work of the institute is most valuable. The institute is, however, of further use to the public, as the secretary keeps a list of well-recommended monthly nurses that can thoroughly be depended upon. Ladies pay a fee of half-a-crown on engaging a nurse, which is reasonable enough. The club-room is open on Friday evenings from six to ten o'clock, when members may bring their friends, and avail themselves of a good medical library and a small collection of light literature. All inquiries should be addressed to the secretary.

---

THE Lord Mayor has very wisely decided not to give the official sanction of the Mansion House to Mr. George Potter's scheme for providing Convalescent Homes for working men. If the working men of East London wish generally to celebrate the coming Jubilee by founding a convalescent home for members of their class, well and good, though we should certainly think it wiser if they decided—say through the Hospital Saturday Fund—to augment their support to existing institutions, rather than to multiply their number, probably to the common detriment, as far as funds are concerned. There seems to be a feeling abroad that a Jubilee memorial must take the form of a brand-new edifice of some sort. To pay off debts on existing institutions, and to fill beds which are tenantless through want of means, are not, to the minds of many, commendable modes for the expression of a national joy. From inquiries made by the Lord Mayor, it appears that, of fifteen convalescent institutions participating in the Hospital Sunday Fund, only 1,100, out of a total of 1,700 beds, were constantly occupied last year through want of funds. The addition, therefore, of another such institution—unless it could be endowed—appears to us a very unwise course.

---

THE Victoria Hospital for Sick Children came in, on Tuesday, for a little welcome help which was not primarily intended for it. Mrs. A. J. Layton had proposed to give a concert in aid of a Jubilee movement in Chelsea. The movement in question fell through, but the arrangements for the concert were so far advanced that Mrs. Layton determined to give it and to devote the proceeds to the Children's Hospital at Chelsea. The name of Mr. Sims Reeves was programmed, and the announcement doubtless drew together a larger audience than would otherwise have been the case. But the great tenor was really indisposed, and confined to his bed. The programme was, however, very ably carried out by Madame Antoinette Sterling, Miss José Sherrington, Mrs. Ellis Cameron, Miss Ida Audain, Miss Beata Frances, Miss Amina Godwin, Mr. H. Beaumont, Mr. A. J. Layton, and Mrs. and Miss Layton. We hope that the concert will have proved as great a success pecuniarily as it certainly was musically.

---

THAT the lot of a Russian citizen is a far from pleasant, and by no means an enviable one, has long been generally admitted, but the recent statement of Professor Kovaleffski, of the Kharkov University, adds a new horror to the conditions of existence amongst the subjects of the Czar. The learned Professor informs us that there are at the least one hundred thousand lunatics in Russia, of whom only one-tenth can be provided for by the existing asylums. Thus, there are no less than ninety thousand acknowledged madmen at large throughout the country, free to work any mischief or commit any crime that may happen to occur to them. Such an idea renders the prospect of residence in an African desert or Indian jungle pleasurable compared with life in Russia. Thousands of lunatics are to be met with everywhere in the country, and they retaliate the ill-treatment they receive by occasionally setting fire to houses, or mutilating children. The lot of the ten thousand lunatics under lock and key is reported to be well-nigh unbearable, owing to the maltreatment they receive at the hands of their rough and cruel attendants. If a patient's friends are able to bribe the keepers, he may be left to himself, but otherwise his life becomes an intolerable burthen.

